# PTSD and comorbid MDD is associated with activation of the right frontoparietal network

**DOI:** 10.1016/j.pscychresns.2023.111630

**Published:** 2023-06

**Authors:** Sheri-Michelle Koopowitz, Heather J. Zar, Dan J. Stein, Jonathan C. Ipser

**Affiliations:** a Department of Psychiatry & Neuroscience Institute, Faculty of Health Sciences, University of Cape Town, Rondebosch, South Africa; b South African Medical Research Council (SAMRC), Unit on Child & Adolescent Health, Cape Town, South Africa; c Department of Paediatrics & Child Health, Red Cross War Memorial Children's Hospital, University of Cape Town, Rondebosch, South Africa; d South African Medical Research Council (SAMRC), Unit on Risk and Resilience in Mental Disorders, Cape Town, South Africa

**Keywords:** Comorbid, fMRI, dlPFC

## Abstract

There is growing evidence of abnormalities in intrinsic functional connectivity (FC) in posttraumatic stress disorder (PTSD) and major depressive disorder (MDD). However, there has been less work on the commonly occurring co-presentation of PTSD and MDD. Characterising intrinsic FC abnormalities in this clinical population is important for understanding how they may contribute towards impairments underpinned by different networks. Participants were mothers enroled in the Drakenstein Child Health Study from Western Cape, South Africa. Mothers between 18 and 50 years of age were recruited and divided into 4 groups: PTSD, MDD, PTSD with MDD, and healthy controls. Participants underwent resting-state fMRI at the 18-month postpartum time point. Functional connectivity within and between higher order cognitive control networks, including the salience, dorsal attention, frontoparietal, and default mode networks were compared across the 4 groups. PTSD with comorbid MDD was associated with greater intrinsic FC within the R FPAR, relative to controls and the mono-diagnostic groups. Intrinsic FC differences were observed within the default mode network for the MDD group. No group differences in connectivity between networks were observed. Differential intrinsic connectivity in participants with comorbidity are consistent with evidence that such individuals have more severe illness and require more robust intervention.

## Highlights

• Four groups of participants: PTSD only, MDD only, PTSD with comorbid MDD, and healthy controls.
• Women from LMIC underwent rest-state scan.
• PTSD with comorbid MDD was associated with greater intrinsic FC within the R FPAR.

## 1 Introduction

Resting-state (RS) functional MRI is a non-invasive technique that investigates the temporal relation of neural activity between brain regions based on fluctuations in the intensity of blood oxygen level dependant (BOLD) signal and allows one to map large-scale intrinsic networks in the brain (Biswal et al., 1995; Buckner et al., 2013; Cole et al., 2010; Greicius et al., 2009; Rosazza and Minati 2011). While the intrinsic functional connectivity (FC) of cognitive control networks (e.g., frontoparietal, dorsal attention, and salience networks) has been explored in posttraumatic stress disorder (PTSD) and major depressive disorder (MDD), little focus has been given to neuroimaging alterations in individuals having both PTSD and comorbid MDD. Meta-analyses indicate that individuals diagnosed with either PTSD or MDD present with altered functional connectivity of intrinsic resting-state networks (Kaiser et al., 2015; Koch et al., 2016; Lee et al., 2012; Scott et al. 2015). Previous resting-state studies of MDD and PTSD have observed abnormalities in networks associated with cognitive control, in particular the default mode network (DMN) (Kaiser et al., 2015; Menon and Uddin 2010). MDD has been associated with reduced connectivity of the salience network (SN), dorsal attention network (DAN), and frontoparietal network (FPAR) (Kaiser et al., 2015). Additionally, the salience network (SN) may be relevant to understanding PTSD, as it is involved in the filtering of task-relevant information (Menon and Desmond 2001), and as such, has been associated with arousal. Therefore, greater functional connectivity within the SN may underlie the hyperarousal symptoms, such as hypervigilance, experienced by individuals with PTSD (American Psychiatric Association 2013). Some evidence also suggests that PTSD is associated with greater connectivity, relative to controls, between the SN and the DMN (Sripada et al., 2012a). In PTSD samples, greater functional within-network FC of midbrain regions such as the hippocampus and amygdala has been found, relative to controls (Rabinak et al., 2011; Sripada et al., 2012b). Further, PTSD is associated with abnormal functional connectivity within the DMN and the salience network, relative to controls (Bluhm et al. 2009; Koch et al., 2016; Sripada et al., 2012a).

In the limited literature, a study examining resting-state fMRI in PTSD and comorbid MDD reported that the comorbid group (*n* = 30) exhibited reduced connectivity within the salience network relative to the PTSD-only group (*n* = 25) (Kennis et al., 2014). This suggests that comorbidity heightens the effects of the individual disorder. The comorbid group exhibited less functional connectivity between nodes of the SN and DMN (the bilateral anterior insula and left hippocampus, respectively) relative to the PTSD only group (Kennis et al., 2014). This represents the opposite pattern of functional connectivity to that previously reported for individuals with a sole diagnosis of PTSD (Sripada et al., 2012a), suggesting qualitative differences between the monodiagnostic group and the comorbid group. Kennis et al. (2014) did not utilise a control group of participants without psychopathology, therefore it is not possible to isolate the effects of PTSD or MDD, or to investigate whether a comorbid diagnosis has either qualitative or quantitative effects (or both) on resting-state functional connectivity of cognitive control networks.

There is a relative paucity of research examining whether resting-state abnormalities in individuals with PTSD and comorbid MDD are qualitatively similar to what is found in either of these disorders, or whether comorbidity of these disorders is associated with a different pattern of connectivity changes. To address the paucity of studies of resting-state FC in participants diagnosed with PTSD and comorbid MDD, the present study aimed to compare the connectivity of cognitive control and default mode networks in participants diagnosed with PTSD, MDD, and PTSD with comorbid MDD. Cognitive control networks of interest included the default mode, salience, dorsal attention, and frontoparietal networks. We expected that the PTSD group would display reduced connectivity within the DMN, relative to controls, in addition to greater connectivity within the SN; while the MDD group would exhibit greater connectivity within the DMN and between the DMN and FPAR and DAN, relative to controls. Based on the limited literature, we expected that the comorbid group would demonstrate abnormal connectivity within the DAN, FPAR, and SN compared to controls, with reduced connectivity within the salience network, relative to PTSD group.

## 2 Methods and materials

### 2.1 Participants

Participants were recruited from the Drakenstein Child Health Study (DCHS) (Zar et al., 2015), from the Western Cape, South Africa. Pregnant women were recruited from two primary health care clinics, Mbekweni (serving a predominantly black African community) and TC Newman (serving a mixed ancestry community). Mothers were enroled in the Drakenstein Child Health Study at 20 to 28 weeks’ gestation while attending routine antenatal care and were prospectively followed. Women were eligible for the study if they were 18 years or older, between 20 and 28 weeks gestation, planned attendance at one of the two recruitment clinics and intended to remain in the area for the next 12 months (Zar et al., 2015). Exclusion criteria for the scanning sub-study included: 1) brain injury or loss of consciousness longer than 30 min, 2) inability to speak English, 3) current/lifetime alcohol and/or substance dependence or abuse, 4) psychiatric illness, including psychosis, other than PTSD and/or MDD, and 5) standard MRI exclusion criteria, such as claustrophobia and presence of ferromagnetic objects in the participant's body. Psychological and physical trauma exposures were not exclusion criteria for the control group; therefore, the control group consisted of trauma-exposed and trauma-naïve controls. A total of 112 participants were recruited for this study.

#### 2.1.1 Procedure

Scanning took place at the 18-month postpartum timepoint, after undergoing neuropsychiatric interviews, conducted by a psychiatrist with extensive experience with this population. Scans took place at the Cape Universities Brain imaging Centre (CUBIC-SUN) at Tygerberg Hospital at Stellenbosch University (3T Magnetom Allegra Syngo, Siemens), and subsequently at the Cape Universities Body Imaging Centre (CUBIC-UCT) at Groote Schuur Hospital at the University of Cape Town (3T Magnetom Skyra, Siemens).

### 2.2 Materials and diagnoses

#### 2.2.1 Diagnoses

Diagnoses were made using DSM-IV criteria using the Mini International Neuropsychiatric Interview (MINI, 5th edition) (Sheehan et al. 1998). If participants had MINI-diagnosed lifetime/current PTSD, the Clinician Administered PTSD Scale (CAPS) (Blake et al., 1995) was administered. Due to the inclusion of lifetime PTSD diagnoses, CAPS assessments were undertaken to ensure that participants with lifetime PTSD experienced moderate to severe PTSD symptoms at the time of scanning. The scoring rule suggested by Weathers et al. (1999) and Orr (1997) was utilised to diagnose PTSD in this sample. This scoring method requires a total CAPS score greater than or equal to 45 to confirm a PTSD diagnosis and has demonstrated reliability against a gold-standard diagnostic instrument (namely, the SCID PTSD module) (Weathers et al., 1999). Where participants did not speak English as their first language, a translator was utilized. Participants completed the Beck Depression Inventory (BDI-II) at the scan visit (Beck et al., 1996).

Women who had a current/lifetime PTSD diagnosis on the MINI and CAPS formed the PTSD group; participants who had a current/lifetime MDD diagnosis on the MINI were part of the MDD group, and participants who had current/lifetime PTSD and MDD on the MINI and CAPS formed the comorbid group. Due to low numbers of current PTSD and MDD, lifetime diagnoses were included to increase sample size and power of analyses to detect group differences. The control group consisted of participants from the DCHS with no current or lifetime psychiatric illness.

### 2.3 Data acquisition

The scan session began with a 10-minute high resolution MPRAGE structural scan which allowed the participants to acclimatise to the scanner and settle. This was followed by an 8-minute resting-state scan, prior to which participants were instructed to keep their eyes open and focus, for the duration of the scan, on a fixation cross projected onto a screen within the scanner bore. The following parameters were used at the CUBIC-UCT scanner, utilising a 32-channel head coil, to obtain resting-state data: TR = 2000 ms, TE = 30 ms, 3.8 × 3.8 × 4.0 mm^3^ voxels, slice thickness = 4.0 mm, flip angle = 77°, FOV = 240 × 240 mm^2^, 30 sagittal slices. The whole-brain multi-echo high resolution T1-weighted gradient echo image sequence acquired at this scanner site employed the following parameters: 128 slices, TR = 2530 ms, TE = 1.69, 3.55, 5.41, 7.27 ms, FOV = 256 × 256 mm^2^, 1.0 × 1.0 × 1.5 mm^3^ voxels, slice thickness = 1.5 mm, inter-slice gap = 1 mm. The resting-state sequence at CUBIC-SUN scanner utilised a four-channel head coil, employed the following parameters: TR = 2000 ms, TE = 30 ms, 3.8 × 3.8 × 4.0 mm^3^ voxels, slice thickness = 4.0 mm, flip angle = 77°, FOV = 240 × 240 mm, 33 sagittal slices. Structural scans at CUBIC-SUN were acquired using the following parameters: 128 slices, TR = 2000 ms, TE = 1.53, 3.21, 4.89, 6.57 ms, FOV = 256 × 256 mm^2^, 1.0 × 1.0 × 1.5 mm^3^, slice thickness = 1.5 mm. Echo planar imaging fieldmaps were acquired immediately prior to the resting-state functional sequences at both scanner sites.

### 2.4 Ethical approval

The DCHS was approved by the Faculty of Health Sciences, Human Research Ethics Committee, University of Cape Town (401/2009) and by the Western Cape Provincial Health Research committee. Mothers provided informed consent at enrolment and were re-consented annually. Participants that were scanned provided consent before they arrived for the scan. Consent was done in the mother's preferred language: English, Afrikaans or isiXhosa. This study was performed in accordance with the Declaration of Helsinki (World Medical Association 2013).

### 2.5 Preprocessing pipeline

The *fMRIPrep* preprocessing pipeline was utilized as it is robust to potential input dataset idiosyncrasies (for example, missing acquisitions) and quality checking of preprocessing results allows transparency (Esteban et al. 2019). What follows is a brief description of the method, as described by Esteban et al. (2019).

Using the custom *fMRIPrep* methodology, the following preprocessing steps were applied: First, a reference volume and its skull-stripped version were generated. Next, a deformation field was estimated, to correct for susceptibility distortions, based on a fieldmap that was co-registered to the BOLD reference. Head-motion parameters (transformation matrices and six corresponding rotation and translation parameters) were estimated with respect to the BOLD reference before spatiotemporal filtering. BOLD runs were subsequently slice-time corrected. The BOLD time-series were resampled onto standard space by applying a single, composite transform to correct for head-motion and susceptibility distortions. Next, non-steady state volumes were removed, and spatial smoothing was applied, with an isotropic, Gaussian kernel of 6 mm FWHM (full-width half-maximum). Following this, motion artifacts were identified by running the ICA AROMA algorithm on the pre-processed BOLD in MNI space time-series (Pruim et al., 2015). Components corresponding to motion were subsequently removed from the BOLD time-series, in a "non-aggressive" manner which preserved common covariance shared between components. Following pre-processing with *fMRIPrep*, a general linear regression model, as implemented by AFNI's 3dDeconvolve (Cox 1996), was run on each participant's RS BOLD dataset. Average white matter and CSF signals were regressed out, as part of this model, utilising subject-specific white matter and CSF masks, as were constant, linear, squared, and cubic trends in the data. A band pass filter (0.01- 0.1 Hz) was applied to the data, as the final step in the processing pipeline. For the remainder of the participants, fieldmap acquisitions were erroneously restricted to phase difference images only. For these participants, the unique functionality afforded by the *fMRIPrep* to estimate a deformation field utilising the participant-specific T1 anatomical sequence was utilised. Based on the estimated susceptibility distortion, an unwarped BOLD reference was calculated for a more accurate co-registration with the anatomical reference (T1w reference).

**Fig. 1:**
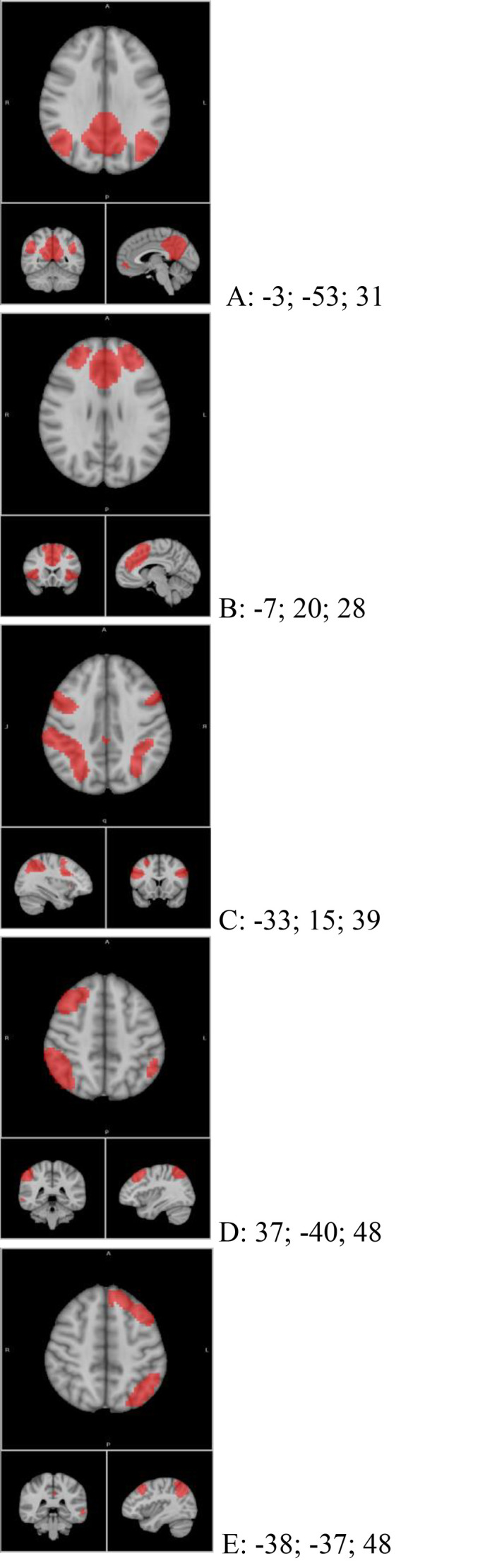
Network masks and their MNI co-ordinates. (a) Default Mode Network (DMN); (b) Salience Network (SN); (c) Dorsal Attention Network (DAN); (d) right Frontoparietal Network (RFPAR); (e) left Frontoparietal Network (LFPAR). MNI co-ordinates printed above each network.

To assess network connectivity, masks were created for networks of interest (left and right frontoparietal, default mode, dorsal attention, and salience networks) based on components extracted using group ICA. For this purpose, FSL's melodic tool (Jenkinson et al., 2012) was applied to the smoothed output of ICA-AROMA, concatenated across the 28 healthy control participants, with dimensionality set to 25 independent components (ICs). Networks were visually inspected and identified by independent raters, with reference to networks published in Allen et al. (2011) and Smith et al. (2009). Network masks were created by thresholding voxels in each IC at *Z* = 4 and restricting clusters to those containing at least 100 2 mm isotropic voxels (see Fig. 1). Seeds for masks were created by centring spheres of 6 mm radius at the peak voxels in each of the clusters. Hemisphere-specific seeds were created from the single SN network cluster that extended across the bilateral insula and basal ganglia by identifying localised peak voxels corresponding to the right and left anterior insula. A summary of network seeds and peak voxel co-ordinates can be found in Table 1.

**Table 1:** Peak voxel co-ordinates for network seeds.

Network	Seed	Peak co-ordinates (x, y, z)	Region
DMN	1	-0.5; +57.5; +3.5	L & R anterior cingulate
	2	-0.5; -52.5 + 31.5	L & R precuneus
	3	+47.5; -68.5; +31.5	R angular gyrus
	4	-40.5; -78.5; +39.5	L precuneus
	5	+11.5; -56.5; +15.5	R posterior cingulate
	6	-28.5; -38.5; -10.5	L hippocampal gyrus
	7	+25.5; -34.5; -12.5	R hippocampal gyrus
	8	+7.5; -70.5; +35.5	R precuneus
R FPAR	1	+45.5; +23.5; +41.5	R dlPFC
	2	+55.5; -52.5; +45.5	R inferior parietal
	3	+65.5; -36.5; -4.5	R middle temporal gyrus
	4	-56.5; -54.5; +45.5	L inferior parietal
	5	+3.5; -36.5; +43.5	L & R cingulate gyrus
	6	+45.5; +55.5; -2.5	R middle frontal
L FPAR	1	-42.5; +9.5; +51.5	L middle frontal gyrus
	2	-54.5; -60.5; +33.5	L supramarginal gyrus
	3	-52.5; +23.5; -4.5	L inferior frontal gyrus
	4	-62.5; -34.5; -2.5	L middle temporal gyrus
	5	+23.5; -74.5; -22.5	R declive, cerebellum
	6	-4.5; -50.5; +33.5	L precuneus
	7	+55.5; -58.5; +41.5	R inferior parietal lobule
Salience	1	+11.5; +1.5; +15.5	basal ganglia/insula
	2	+33.0; +28.0; +1.0	R anterior insula
	3	-35.0; +27.0; -1.0	L anterior insula
	4	+1.5; +15.5; +49.5	dorsal ACC
	5	+1.5; -42.5; +5.5	posterior cingulate
	6	+41.5; -0.5; +55.5	R middle frontal
DAN	1	-46.5; +9.5; +31.5	L inferior/middle frontal gyrus/dlPFC
	2	-30.5; -70.5; +43.5	L precuneus
	3	+47.5; +37.5; +19.5	R inferior frontal gyrus/dlPFC
	4	+31.5; -66.5; +43.5	R precuneus
	5	-60.5; -58.5; -8.5	L inferior temporal gyrus
	6	-57.5; -52.5; -8.5	R middle temporal gyrus
	7	-2.5; -34.5; +39.5	L cingulate gyrus
	8	-36.5; +37.5; -12.5	L middle frontal gyrus
	9	+31.5; +39.5; -12.5	R middle frontal gyrus

### 2.6 Data analysis

ANOVAs and t-tests were run to examine group differences on demographic variables, using SPSS 25. For each of the DMN, SN, DAN, right frontoparietal, and left frontoparietal networks, FC matrices were computed amongst pairs of the average time-series of each of the seeds, using AFNI's 3dNetCorr. Group differences in connectivity were identified with multivariate models (MVMs) created using AFNI's FATCAT (Taylor et al., 2016; Taylor and Saad 2013), a suite of tools that provides an intuitive interface to AFNI's 3dMVM program (Chen et al., 2014). This methodology results in a hierarchical analysis: first, an omnibus F-stat provides a measure of the model at a “network level”, including all ROIs; if the effect of interest is significant, then a follow-up investigation occurs at the “ROI level”, by using post hoc t-tests to evaluate the same model for each ROI. In this way, if a significant network-level effect is observed, one can use the ROI level results to determine which part(s) of the network are predominantly responsible, or whether the effect is dispersed. An example of the 3dMVM model syntax generated by the FATCAT toolbox is provided as supplementary material (Online resource 1).

Five separate models were run, one for each network, to test group differences in connectivity between seeds constituting the network. In addition, a BOLD time-series for each network was obtained by averaging the time-series for seeds within each of the networks, with these global network time-series used as input to a multivariate model of group differences in between-network connectivity estimates.

Since two data acquisition sites were utilised in this study, “scanner” was initially included as a covariate in all of the models (within-network and between-network), to check for effects due to acquisition differences. If there was no scanner effect for a specific model, the scanner covariate was removed from the model, and it was run again. If there was a significant scanner effect, we ran a subsequent model in which we removed pairwise comparisons between seeds within a network where significant differences in connectivity as a function of scanner site were observed (at *p* < .1).

Secondary analyses included running models to test for total CAPS score interactions and total BDI score with network connectivity (both within and between). The first model checked for scanner effects. If there was no scanner effect, the next model included a group and CAPS or BDI total score interaction term. If this term was not significant, the model was run again to test for a CAPS or BDI total score main effect.

## 3 Results

### 3.1 Participants

Of the 112 participants recruited for this study, *n* = 8 were excluded from the analyses due to: incomplete diagnostic information (*n* = 3), subthreshold PTSD diagnosis (*n* = 1), presence of alcohol abuse (*n* = 1), and poor quality anatomical T1 dataset (*n* *=* 3). A further 19 participants were removed after initial analysis, due to excessive motion during the fMRI scan, operationalised as those for whom there was less than 5 min of scanning data during which displacement was always < 0.3 mm. Thus, a total of 85 participants were included in the final analyses. Group sizes were as follows: PTSD *n* = 22 (*n* = 2 current PTSD); MDD *n* = 18 (*n* = 2 current MDD); PTSD+MDD *n* = 17 (*n* = 1 current PTSD; *n* = 1 current MDD); control *n* = 28. The descriptive statistics for the groups can be found in Table 2.

**Table 2:** Sample demographics.

	PTSD	MDD	PTSD+MDD	Control		
	*n* = 22	*n* = 18	*n* = 17	*n* = 28	*F/t*	*p* value
Age *(SD)*^a^	31.1 *(8.1)*	28.2 *(7.6)*	30.4 *(7.1)*	26.1 *(6.2)*	2.34	.08
Education *(SD)*^b^	10.7 *(2.9)*	10.9 *(1.8)*	10.2 *(1.9)*	11.4 *(1.8)*	1.01	.39
BDI-II total score	12.68 *(11.86)*	14.72 *(11.70)*	23.53 *(17.94)*	6.78 *(8.12)*	6.52	<0.001
CAPS total severity score c	69.1 *(18.9)*	NA	89.4 *(15.3)*	NA	3.62	.001

*Note.* PTSD = PTSD only group; MDD = Depression only group; PTSD+MDD = comorbid group; BDI-II = Beck Depression Inventory II; CAPS = Clinician Administered PTSD Scale

a based on 85 participants

b based on 77 participants.

c based on 39 participants; means are presented with standard deviations presented in parentheses.

### 3.2 Descriptive statistics

No significant differences were observed between any of the groups on either age (*F* (3, 81) = 2.34, *p* = .08) or education (*F* (3, 74)= 1.01, *p* = .39). CAPS scores were significantly greater on average in the comorbid group than the PTSD group (*M* = 89.4, *SD* = 15.3 versus *M* = 69.1, *SD* = 18.9, respectively, *t*(37) = -3.62, *p* = .001, two-tailed). There was a significant group difference on BDI scores (*F* (3, 80) = 6.52, *p* < .001). Post hoc comparisons using Tukey's HSD indicated that controls (*M* = 6.78, *SD* = 8.12) significantly differed from the comorbid group (*M* = 23.53, *SD* = 17.94); and the comorbid group differed from the PTSD group (*M* = 12.68, *SD* = 11.86).

### 3.3 Primary analyses - within-network connectivity

#### 3.3.1 DMN

There was no scanner effect (χ^2^ = 2.4, *p* = .12), and therefore scanner was omitted from a model including all pairwise connectivity estimates for all DMN ROIs. The final model indicated that there were significant network connectivity differences with group (χ^2^ = 5.65, *p* = .017), and post hoc pairwise group comparisons indicated that differences in connectivity were primarily observed for the MDD group, with participants displaying greater FC relative to the other groups. ROIs where connectivity differences, with a variety of regions, were most apparent included the ACC, precuneus, and hippocampal gyrus. Average correlations for these ROI-pairs, stratified by group, can be seen in Table 3.

**Table 3:** Average correlations for DMN seed-pairs where intrinsic connectivity was moderated by group.

Seed-pair	Control	Comorbid	MDD	PTSD	Difference	*t* statistic	*p* value
ACC - left precuneus	.38 *(0.31)*	.22 *(0.28)*	.34 *(0.34)*	.43 *(0.26)*	Comorbid < CTRLS	2.23	.028
					Comorbid < PTSD	-2.29	.025
ACC - left hippocampal gyrus	.13 *(0.26)*	.19 *(0.16)*	.29 *(0.31)*	.3 *(0.21)*	CTRLS < MDD	-2.03	.045
					CTRLS < PTSD	-2.28	.025
Precuneus - left hippocampal gyrus	.58 *(0.28)*	.4 *(0.25)*	.51 *(0.36)*	.55 *(0.22)*	Comorbid < CTRLS	2.61	.01
Right angular gyrus - left hippocampal gyrus	.31 *(0.28)*	.44 *(0.25)*	.53 *(0.16)*	.41 *(0.25)*	CTRLS < MDD	-2.47	.015
Right angular gyrus - right hippocampal gyrus	.29 *(0.23)*	.39 *(0.17)*	.46 *(0.21)*	.31 *(0.27)*	CTRLS < MDD	-2.33	.022
Left precuneus - left hippocampal gyrus	.25 *(0.32)*	.42 *(0.24)*	.5 *(0.22)*	.42 *(0.26)*	CTRLS < MDD	-2.72	.007
Left precuneus - right precuneus	.4 *(0.24)*	.4 *(0.17)*	.51 *(0.21)*	.38 *(0.24)*	PTSD < MDD	2.06	.042
Right posterior cingulate - right hippocampal gyrus	.4 *(0.24)*	.43 *(0.23)*	.56 *(0.17)*	.48 *(0.26)*	CTRLS < MDD	-2.37	.019
Right posterior cingulate - right precuneus	.49 *(0.21)*	.57 *(0.12)*	.67 *(0.12)*	.57 *(0.19)*	CTRL < MDD	-3.09	.027

#### 3.3.2 SN

A scanner effect was detected (χ^2^ = 5.9, *p* = .015), therefore scanner and two seed-pairs (insula and right middle frontal gyrus; right anterior insula and left anterior insula) were removed from the final model. The final model did not show a significant group effect (*F* = 1.11, *p* = .29).

#### 3.3.3 DAN

Scanner produced a significant effect (χ^2^ = 6.1, *p* = .014) in the overall model. Subsequently, the seven seed-pairs for which post-hoc tests indicated that scanner was a significant effect modifier were removed from the final model (left dlPFC and right dlPFC; left dlPFC and left inferior temporal gyrus; left dlPFC and left middle frontal gyrus; left precuneus and left cingulate gyrus; right dlPFC and left middle frontal gyrus; right middle temporal gyrus and left cingulate gyrus; left middle frontal gyrus and right middle frontal gyrus). The final model did not show a significant group effect, χ^2^ = 0.75, *p* = .38.

#### 3.3.4 R FPAR

There was no scanner effect (χ^2^ = 2.42, *p* = .12), therefore the final model included all pairwise connectivity estimates. The final model indicated that there was a significant association with group (χ^2^ = 4.4, *p* = .036). Post hoc pairwise comparisons indicated that differences were observed primarily for the comorbid group, relative to all the other groups, in seed-pairs with increased R FPAR connectivity with the dlPFC and inferior parietal regions. These differences can be seen for each seed-pair in Table 4.

**Table 4:** Average correlations for R FPAR seed-pairs where intrinsic connectivity was moderated by group .

Seed-pair	Control	Comorbid	MDD	PTSD	Difference	*t* statistic	*p* value
Right dlPFC - right inferior parietal	.44 *(0.33)*	.55 *(0.26)*	.32 *(0.21)*	.31 *(0.34)*	MDD < Comorbid	2.43	.017
					PTSD < Comorbid	2.6	.011
Right dlPFC - right middle frontal	.19 *(0.3)*	.39 *(0.36)*	.17 *(0.25)*	.26 *(0.27)*	CTRLS < Comorbid	-2.07	.041
Right inferior parietal - right middle temporal gyrus	.27 *(0.34)*	.47 *(0.33)*	.19 *(0.21)*	.29 *(0.38)*	MDD < Comorbid	2.64	.009
Right inferior parietal - left inferior parietal	.53 *(0.22)*	.56 *(0.22)*	.38 *(0.25)*	.41 *(0.28)*	MDD < CTRLS	2.3	.024
					PTSD < CTRLS	2.11	.038
					MDD < Comorbid	2.1	.039
Right inferior parietal - left, right cingulate gyrus	.34 *(0.29)*	.49 *(0.25)*	.32 *(0.13)*	.35 *(0.29)*	MDD < Comorbid	2.21	.029
Right middle temporal gyrus - left inferior parietal	.23 *(0.25)*	.39 *(0.18)*	.16 *(0.29)*	.27 *(0.26)*	CTRLS < Comorbid	-2.09	.039
					MDD < Comorbid	2.71	.008
Left inferior parietal - left, right cingulate gyrus	.27 *(0.22)*	.41 *(0.25)*	.15 *(0.3)*	.27 *(0.23)*	MDD < Comorbid	2.98	< 0.001

#### 3.3.5 L FPAR

A scanner effect was detected (χ^2^ = 4.61, *p* = .032), therefore the final model excluded seed-pairs that were dependant on scanner (left middle frontal gyrus and right cerebellum; left supramarginal gyrus and right cerebellum; left precuneus and right inferior parietal lobule). The final model failed to detect a significant group effect. A trend effect (χ^2^ = 3.83, *p* = .0504) was observed within the L FPAR network in MDD participants, relative to the other groups. Post hoc comparisons indicated that reductions were observed for the MDD group in seed-pairs that included connections with the middle frontal gyrus and the inferior frontal gyrus. Average correlations for these regions can be found in Table 5.

**Table 5:** Average correlations for L FPAR seed-pairs where intrinsic connectivity was moderated by group.

Seed-pair	Control	Comorbid	MDD	PTSD	Difference	*t* statistic	*p* value
Left middle frontal gyrus - left middle temporal gyrus	.489 *(0.22)*	.38 *(0.35)*	.2 *(0.24)*	.41 *(0.25)*	MDD < CTRLS	3.65	< 0.001
					MDD < Comorbid	2.1	.038
					MDD < PTSD	-2.47	.015
Left middle frontal gyrus - left precuneus	.3 *(0.25)*	.19 *(0.29)*	.11 *(0.18)*	.27 *(0.24)*	MDD < CTRLS	2.43	.017
					MDD < PTSD	-2.01	.047
Left middle frontal gyrus - right inferior parietal lobule	.33 *(0.24)*	.27 *(0.24)*	.07 *(0.24)*	.17 *(0.22)*	MDD < CTRLS	3.43	< 0.001
					PTSD < CTRLS	2.31	.023
					MDD < Comorbid	2.31	< 0.001
Left inferior frontal gyrus - left precuneus	.05 *(0.26)*	.09 *(0.25)*	-0.06 *(0.32)*	.11 *(0.19)*	MDD < Comorbid	2.12	.036
					MDD < PTSD	-2.43	.016
Left inferior frontal gyrus - right inferior parietal lobule	.13 *(0.24)*	.07 *(0.35)*	-0.028 *(0.26)*	.15 *(0.19)*	MDD < CTRLS	2.01	.047
					MDD < PTSD	-2.15	.033

#### 3.3.6 Primary analyses - between-network connectivity

No scanner effect was observed (χ^2^ = 2.78, *p* = .095), therefore all between-network connectivity estimates were included in the final model. No significant group effect was observed for between-network connectivity (χ^2^ = 0.78, *p* = .38).

### 3.4 Secondary analyses - CAPS total score models – within-network connectivity

#### 3.4.1 DMN

No effect of scanner was observed in the model of the association of CAPS score with DMN connectivity (χ^2^ = 3.49, *p* = .06). The second model indicated that there was no significant interaction between group and total CAPS score (χ^2^ = 0.34, *p* = .56), therefore the analysis was run without the interaction term. The final model indicated that there was a significant effect for CAPS total score on DMN connectivity (χ^2^ **=** 4.5, *p* = .038). Post hoc analysis indicated that higher CAPS scores were associated with greater connectivity between the precuneus and the right posterior cingulate seeds (*p* = .01).

#### 3.4.2 R FPAR

No scanner effect was observed in the model of association of CAPS score and R FPAR connectivity (χ^2^ = 0.69, *p* = .4). The second model indicated that there was no significant group and CAPS total score interaction (χ^2^ = 1.29, *p* = .25), therefore the analysis was run without the interaction term. The final model indicated that there was no effect of CAPS total score on right frontoparietal connectivity (χ^2^ = 0.52, *p* = .47).

#### 3.4.3 L FPAR

No scanner effect was detected in the first model (χ^2^ = 3.49, *p* = .062), and the second model indicated that there was an interaction for group and CAPS total score (χ^2^ = 7.1, *p* = .008). Post hoc analysis did not indicate significant pairwise interaction effects for any seed-pairs, however a number of subthreshold effects were observed. Post hoc analysis indicated that the comorbid group, relative to the PTSD group, exhibited greater subthreshold connectivity effects for the left precuneus.

#### 3.4.4 SN

A significant scanner effect was observed (χ^2^ = 5.38, *p* = .02), and two seed-pairs (right anterior insula – left anterior insula; right anterior insula – posterior cingulate) were removed from the subsequent analyses. There was no significant interaction between group and CAPS total score (χ^2^ = 0.87, *p* = .35), therefore the interaction term was removed from the model. The final model indicated that there was no effect of CAPS on salience network connectivity (χ^2^ = 1.569, *p* = .21).

#### 3.4.5 DAN

No scanner effect was observed (χ^2^ = 2.22, *p* = .14) and there was no significant interaction between CAPS total score and group (χ^2^ = 0.58, *p* = .48), therefore the interaction term was removed from the final model. The final model indicated that there was no significant association between CAPS total score and dorsal attention network connectivity (χ^2^ = 1.41, *p* = .23).

#### 3.4.6 Secondary analyses – CAPS total score models - between-network connectivity

No scanner effect was observed in the model of association of CAPS score and between-network connectivity (χ^2^ = 0.146, *p* = .7). The interaction term in the model was not significant (χ^2^ = 1.97, *p* = .16); therefore, it was removed from the final model. The final model indicated that there was no significant association between CAPS total score and between-network connectivity in this sample (χ^2^ = 1.06, *p* = .3).

### 3.5 Secondary analyses - BDI total score models – within-network connectivity

#### 3.5.1 DMN

A significant effect of scanner on DMN within-network connectivity was detected (χ^2^ = 4.519, *p* = .034), and three seed-pairs (posterior cingulate - right angular gyrus; right precuneus - left parahippocampal gyrus; left parahippocampal gyrus - right parahippocampal gyrus) were removed from the subsequent analyses. The interaction term in the model was not significant (χ^2^ = 1.00, *p* = .32); therefore, it was removed from the final model. The final model indicated that there was no significant association between BDI total score and DMN within-network connectivity in this sample (χ^2^ = 1.10, *p* = .29).

#### 3.5.2 R FPAR

A significant effect of scanner on right FPAR within-network connectivity was detected (χ^2^ = 7.323, *p* = .007), and three seed-pairs (right middle frontal gyrus - right inferior parietal; left inferior parietal - left inferior parietal; right middle temporal gyrus - left cingulate gyrus) were removed from the subsequent analyses. The interaction term in the model was not significant (χ^2^ = 3.445, *p* = .063); therefore, it was removed from the final model. The final model indicated that there was no significant association between BDI total score and DMN within-network connectivity in this sample (χ^2^ = 2.85, *p* = .09).

#### 3.5.3 L FPAR

A significant effect of scanner on left FPAR within-network connectivity was detected (χ^2^ = 10.078, *p* = .002), and four seed-pairs (left middle frontal gyrus - left middle temporal gyrus; left middle frontal gyrus - right inferior parietal lobule; left angular gyrus - left posterior cingulate cortex; left middle frontal gyrus - right inferior parietal lobule) were removed from the subsequent analyses. The interaction term in the model was significant (χ^2^ = 9.562, *p* = .002). The association between BDI total score and connectivity was moderated by group for 9 seed pairs. For four seed pairs, higher BDI score was associated with less connectivity in the comorbid than the PTSD-only group, with connectivity between the left middle frontal gyrus (to both the left angular gyrus and left posterior cingulate) and the left inferior frontal gyrus (to both the left angular gyrus and left posterior cingulate) affected. Higher BDI scores were associated with greater connectivity for the comorbid group, on the other hand, between the right cerebellum and the (1) left posterior cingulate cortex (relative to MDD-only subjects) and the (2) right inferior parietal cortex (relative to the control group). Higher BDI total scores predicted greater connectivity between the right cerebellum and the left angular gyrus, as well as between the left posterior cingulate cortex and right inferior parietal lobule, in PTSD-only than MDD-only groups, and in PTSD-only patients compared to controls for the left inferior frontal gyrus and right inferior parietal lobule

#### 3.5.4 SN

A significant effect of scanner on SN within-network connectivity was detected (χ^2^ = 6.946, *p* = .008), and five seed-pairs (right caudate - right posterior cingulate cortex; right insula, ant. - left inferior frontal gyrus; right insula, ant – SMA; left inferior frontal gyrus – SMA; SMA - right middle frontal gyrus) were removed from the subsequent analyses. The interaction term in the model was significant (χ^2^ = 3.888, *p* = .049). All interaction effects implicated greater connectivity with higher total BDI scores for the PTSD-only group in seed pairs involving the supplementary motor area (SMA). This effect was observed for the right caudate, relative to the comorbid group, as well as the right posterior cingulate cortex, compared to all other groups.

#### 3.5.5 DAN

No scanner effect was observed in the model of association of BDI score and DAN connectivity (χ^2^ = 0.89, *p* = .35). The second model indicated that there was no significant group and BDI total score interaction (χ^2^ = 0.76, *p* = .38), therefore the analysis was run without the interaction term. The final model indicated that there was no effect of BDI total score on dorsal attention network connectivity (χ^2^ = 1.28, *p* = .26).

#### 3.5.6 Secondary analyses – BDI total score models - between-network connectivity

A significant effect of scanner on between-network connectivity was detected (χ^2^ = 6.123, *p* = .013), and one seed-pair (right frontoparietal – left frontoparietal) were removed from the subsequent analyses. The interaction term in the model was not significant (χ^2^ = 1.36, *p* = .24); therefore, it was removed from the final model. The final model indicated that there was no significant association between BDI total score and between-network connectivity in this sample (χ^2^ = 1.89, *p* = .17).

## 4 Discussion

In this study, PTSD with comorbid MDD was associated with greater intrinsic functional connectivity within the R FPAR, relative to controls and the mono-diagnostic groups. We also observed hyperconnectivity within the DMN for the MDD group. We did not observe significant between-network connectivity for any of the network estimates.

Greater functional connectivity within the R FPAR for participants diagnosed with PTSD and comorbid MDD has not been reported in the literature, to date. Within this network, the comorbid group exhibited greater functional connectivity between frontal region seed-pairs that are typically involved with higher order neurocognitive functions, such as set-switching, planning, and decision-making (Elliott 2003). Given evidence that the R FPAR regulates and co-ordinates task dependant switching (Gao and Lin 2012), and has the most temporal variability of the cognitive control networks (Gonzalez-Castillo et al., 2014), this finding suggests that PTSD with comorbid MDD may be associated with abnormal executive functioning and increased symptom severity. Therefore, greater connectivity within the R FPAR may indicate less temporal variability over the 8-minute scan sequence in the participants diagnosed with both PTSD and MDD, and reduced flexibility and ability to adapt to task demands in these participants. Further studies that test dynamic patterns of connectivity within this network will be able to test this hypothesis.

In this cohort, MDD was associated with abnormal intrinsic functional connectivity within the DMN, which is consistent with current literature and meta-analyses (see Greicius et al., 2007; Kaiser et al., 2015). Findings from previous research has reported that DMN hyperconnectivity, particularly within the hippocampal region, relates to greater levels of rumination and self-referential thought; commonly observed in individuals with MDD (Cavanna and Trimble 2006; Ho et al. 2019; Kaiser et al., 2015).

Our secondary analyses, which examined the association of symptom severity with resting-state network connectivity, determined that PTSD symptom severity was associated with altered DMN connectivity. Depression symptom severity was associated with altered connectivity of the L FPAR and salience network. Notably, participants in the comorbid group with higher depression scores demonstrate less connectivity of frontal cognitive control regions in the L FPAR, despite the observation that on average corresponding regions in the contralateral network are more strongly connected in this group in our sample. The restriction of this finding to the comorbid group may partly reflect a confound introduced by range restrictions in the data, given the observation that BDI total scores for the comorbid group were substantially higher than for the other groups (including the MDD group – see Table 2). We were unable to replicate the commonly reported finding that depression severity moderates DMN connectivity (Kaiser et al., 2015). These findings indicate that the comorbid group demonstrates greater abnormal intrinsic connectivity relative to the PTSD, MDD, and control groups, indicating that the addition of a comorbid diagnosis impairs intrinsic functional connectivity.

A range of reasons may be proposed for our inability to replicate previous functional connectivity findings. In particular, there may be important clinical differences across study populations. While the present study sample was comprised of female civilians from a low-middle income region of the Western Cape and exposed to multiple trauma types, the majority of published articles, particularly PTSD articles, have been conducted on male veterans in USA or Europe, with a single trauma type (Danckwerts and Leathem 2003).

Furthermore, several limitations to this study should be emphasized. First, in line with the previous paragraph, our use of a female low-income sample may limit generalizability of findings. Second, due to reasons beyond the control of the study, two separate scanners were used during this study. Group differences were only observed for those networks where scanner effects were not detected, and for which we were able to run tests using the complete connectivity matrix for seeds constituting those networks. Although regarded as necessary to guard against bias introduced by these scanner effects, it is possible that removal of connectivity estimates from those models where scanner effects were observed may have affected our power to detect group differences. Third, rumination symptoms were not measured in this study. As a result, we were not able to fully examine any association rumination symptoms might have in our sample with the functional connectivity of the DMN.

In conclusion, this study provides novel evidence suggesting that PTSD with comorbid MDD is associated with greater within-network connectivity in the right frontoparietal network, relative to mono-diagnostic groups and controls. In addition, we were able to add to the growing body of evidence for disturbances within the DMN in individuals diagnosed with MDD. No evidence was, however, observed for an association of either PTSD and/or MDD on intrinsic functional connectivity between cognitive control networks, however. Future utilisation of resting-state FMRI in combination with other neuroimaging modalities (such as structural imaging) may give us a better understanding of the biological basis for comorbidity of psychiatric disorders, such as PTSD together with MDD, and informed targeted interventions for their treatment.

## Funding

The study is funded by the Bill and Melinda Gates Foundation (OPP 1017641). Additional support for HJZ and DJS was provided by the Medical Research Council of South Africa

## Authors’ contributions

All authors contributed to the study conception and design (DJS; HJZ; JI; SK). Material preparation, data collection and analysis were performed by Sheri-Michelle Koopowitz and Jonathan Ipser. The first draft of the manuscript was written by Sheri-Michelle Koopowitz, and all authors commented and edited the manuscript. All authors read and approved the final manuscript.

## Ethics approval

All procedures performed were in accordance with the ethical standards of the institutional and with the 1964 Helsinki Declaration and its later amendments or comparable ethical standards. The DCHS was approved by the Faculty of Health Sciences, Human Research Ethics Committee, University of Cape Town (401/2009) and by the Western Cape Provincial Health Research committee.

## Consent to participate

Informed consent was obtained from all participants at enrolment and were re-consented annually. Consent was done in the participant's preferred language: English, Afrikaans or isiXhosa.

## Consent for publication

Not applicable

## Code availability

Not applicable

## Declaration of Competing Interest

The authors declare that there are no conflicts of interests

## Appendix Supplementary materials

Image, application 1
