# Supplementary material for: PTSD and comorbid MDD is associated with activation of the right frontoparietal network
Source: Psychiatry Res. 2023 Jun;331:111630. doi: 10.1016/j.pscychresns.2023.111630 (PMC10172683; doi:10.1016/j.pscychresns.2023.111630)
Supplement: Supplementary file 1 [file mmc1.docx]

**PTSD and comorbid MDD is associated with activation of the right frontoparietal network**

Sheri-Michelle Koopowitz^a*^, Heather J. Zar^b, c^, Dan J. Stein^a, d^, & Jonathan C. Ipser^a^

^a^ Department of Psychiatry & Neuroscience Institute, Faculty of Health Sciences, University of Cape Town, Rondebosch, South Africa

^b^ South African Medical Research Council (SAMRC), Unit on Child & Adolescent Health, Cape Town, South Africa

^c^ Department of Paediatrics & Child Health, Red Cross War Memorial Children’s Hospital, University of Cape Town, Rondebosch, South Africa

^d^ South African Medical Research Council (SAMRC), Unit on Risk and Resilience in Mental Disorders, Cape Town, South Africa

*Corresponding author: Sheri Koopowitz

[S.Koopowitz@](mailto:S.Koopowitz@)uct.ac.za

**Supplementary material**

Example 3dMVM script generated using the AFNI FATCAT toolbox. Model of between network differences in connectivity between groups, after adjusting for the effects of age and motion.

# Made_by_command :

# fat_mvm_scripter.py --vars='group age avgmot' --prefix='group_w_age_avgmot_script' --log_file='group_w_age_avgmot_MVMprep.log' --table='group_w_age_avgmot_MVMtbl.txt'

#

# ROI_list : DAN__LFPAR DAN__RFPAR DAN__SAL DMN__DAN DMN__LFPAR DMN__RFPAR DMN__SAL RFPAR__LFPAR SAL__LFPAR SAL__RFPAR

# Parameter_list : CC FZ

# Qvar_list : age avgmot

# Cvar_list : group( ctrl dual mdd ptsd )

# Ivar_list :

3dMVM -prefix group_w_age_avgmot_script_MVM \

-bsVars "group+age+avgmot" \

-wsVars "ROI" \

-qVars "age,avgmot" \

-num_glt 120 \

-gltLabel 1 DAN__LFPAR--group\(+ctrl-dual\) -gltCode 1 "ROI : 1*DAN__LFPAR group : 1*ctrl -1*dual" \

-gltLabel 2 DAN__LFPAR--group\(+ctrl-mdd\) -gltCode 2 "ROI : 1*DAN__LFPAR group : 1*ctrl -1*mdd" \

-gltLabel 3 DAN__LFPAR--group\(+ctrl-ptsd\) -gltCode 3 "ROI : 1*DAN__LFPAR group : 1*ctrl -1*ptsd" \

-gltLabel 4 DAN__LFPAR--group\(+dual-mdd\) -gltCode 4 "ROI : 1*DAN__LFPAR group : 1*dual -1*mdd" \

-gltLabel 5 DAN__LFPAR--group\(+dual-ptsd\) -gltCode 5 "ROI : 1*DAN__LFPAR group : 1*dual -1*ptsd" \

-gltLabel 6 DAN__LFPAR--group\(+mdd-ptsd\) -gltCode 6 "ROI : 1*DAN__LFPAR group : 1*mdd -1*ptsd" \

-gltLabel 7 DAN__LFPAR--group^^ctrl -gltCode 7 "ROI : 1*DAN__LFPAR group : 1*ctrl " \

-gltLabel 8 DAN__LFPAR--group^^dual -gltCode 8 "ROI : 1*DAN__LFPAR group : 1*dual " \

-gltLabel 9 DAN__LFPAR--group^^mdd -gltCode 9 "ROI : 1*DAN__LFPAR group : 1*mdd " \

-gltLabel 10 DAN__LFPAR--group^^ptsd -gltCode 10 "ROI : 1*DAN__LFPAR group : 1*ptsd " \

-gltLabel 11 DAN__LFPAR--age -gltCode 11 "ROI : 1*DAN__LFPAR age : " \

-gltLabel 12 DAN__LFPAR--avgmot -gltCode 12 "ROI : 1*DAN__LFPAR avgmot : " \

-gltLabel 13 DAN__RFPAR--group\(+ctrl-dual\) -gltCode 13 "ROI : 1*DAN__RFPAR group : 1*ctrl -1*dual" \

-gltLabel 14 DAN__RFPAR--group\(+ctrl-mdd\) -gltCode 14 "ROI : 1*DAN__RFPAR group : 1*ctrl -1*mdd" \

-gltLabel 15 DAN__RFPAR--group\(+ctrl-ptsd\) -gltCode 15 "ROI : 1*DAN__RFPAR group : 1*ctrl -1*ptsd" \

-gltLabel 16 DAN__RFPAR--group\(+dual-mdd\) -gltCode 16 "ROI : 1*DAN__RFPAR group : 1*dual -1*mdd" \

-gltLabel 17 DAN__RFPAR--group\(+dual-ptsd\) -gltCode 17 "ROI : 1*DAN__RFPAR group : 1*dual -1*ptsd" \

-gltLabel 18 DAN__RFPAR--group\(+mdd-ptsd\) -gltCode 18 "ROI : 1*DAN__RFPAR group : 1*mdd -1*ptsd" \

-gltLabel 19 DAN__RFPAR--group^^ctrl -gltCode 19 "ROI : 1*DAN__RFPAR group : 1*ctrl " \

-gltLabel 20 DAN__RFPAR--group^^dual -gltCode 20 "ROI : 1*DAN__RFPAR group : 1*dual " \

-gltLabel 21 DAN__RFPAR--group^^mdd -gltCode 21 "ROI : 1*DAN__RFPAR group : 1*mdd " \

-gltLabel 22 DAN__RFPAR--group^^ptsd -gltCode 22 "ROI : 1*DAN__RFPAR group : 1*ptsd " \

-gltLabel 23 DAN__RFPAR--age -gltCode 23 "ROI : 1*DAN__RFPAR age : " \

-gltLabel 24 DAN__RFPAR--avgmot -gltCode 24 "ROI : 1*DAN__RFPAR avgmot : " \

-gltLabel 25 DAN__SAL--group\(+ctrl-dual\) -gltCode 25 "ROI : 1*DAN__SAL group : 1*ctrl -1*dual" \

-gltLabel 26 DAN__SAL--group\(+ctrl-mdd\) -gltCode 26 "ROI : 1*DAN__SAL group : 1*ctrl -1*mdd" \

-gltLabel 27 DAN__SAL--group\(+ctrl-ptsd\) -gltCode 27 "ROI : 1*DAN__SAL group : 1*ctrl -1*ptsd" \

-gltLabel 28 DAN__SAL--group\(+dual-mdd\) -gltCode 28 "ROI : 1*DAN__SAL group : 1*dual -1*mdd" \

-gltLabel 29 DAN__SAL--group\(+dual-ptsd\) -gltCode 29 "ROI : 1*DAN__SAL group : 1*dual -1*ptsd" \

-gltLabel 30 DAN__SAL--group\(+mdd-ptsd\) -gltCode 30 "ROI : 1*DAN__SAL group : 1*mdd -1*ptsd" \

-gltLabel 31 DAN__SAL--group^^ctrl -gltCode 31 "ROI : 1*DAN__SAL group : 1*ctrl " \

-gltLabel 32 DAN__SAL--group^^dual -gltCode 32 "ROI : 1*DAN__SAL group : 1*dual " \

-gltLabel 33 DAN__SAL--group^^mdd -gltCode 33 "ROI : 1*DAN__SAL group : 1*mdd " \

-gltLabel 34 DAN__SAL--group^^ptsd -gltCode 34 "ROI : 1*DAN__SAL group : 1*ptsd " \

-gltLabel 35 DAN__SAL--age -gltCode 35 "ROI : 1*DAN__SAL age : " \

-gltLabel 36 DAN__SAL--avgmot -gltCode 36 "ROI : 1*DAN__SAL avgmot : " \

-gltLabel 37 DMN__DAN--group\(+ctrl-dual\) -gltCode 37 "ROI : 1*DMN__DAN group : 1*ctrl -1*dual" \

-gltLabel 38 DMN__DAN--group\(+ctrl-mdd\) -gltCode 38 "ROI : 1*DMN__DAN group : 1*ctrl -1*mdd" \

-gltLabel 39 DMN__DAN--group\(+ctrl-ptsd\) -gltCode 39 "ROI : 1*DMN__DAN group : 1*ctrl -1*ptsd" \

-gltLabel 40 DMN__DAN--group\(+dual-mdd\) -gltCode 40 "ROI : 1*DMN__DAN group : 1*dual -1*mdd" \

-gltLabel 41 DMN__DAN--group\(+dual-ptsd\) -gltCode 41 "ROI : 1*DMN__DAN group : 1*dual -1*ptsd" \

-gltLabel 42 DMN__DAN--group\(+mdd-ptsd\) -gltCode 42 "ROI : 1*DMN__DAN group : 1*mdd -1*ptsd" \

-gltLabel 43 DMN__DAN--group^^ctrl -gltCode 43 "ROI : 1*DMN__DAN group : 1*ctrl " \

-gltLabel 44 DMN__DAN--group^^dual -gltCode 44 "ROI : 1*DMN__DAN group : 1*dual " \

-gltLabel 45 DMN__DAN--group^^mdd -gltCode 45 "ROI : 1*DMN__DAN group : 1*mdd " \

-gltLabel 46 DMN__DAN--group^^ptsd -gltCode 46 "ROI : 1*DMN__DAN group : 1*ptsd " \

-gltLabel 47 DMN__DAN--age -gltCode 47 "ROI : 1*DMN__DAN age : " \

-gltLabel 48 DMN__DAN--avgmot -gltCode 48 "ROI : 1*DMN__DAN avgmot : " \

-gltLabel 49 DMN__LFPAR--group\(+ctrl-dual\) -gltCode 49 "ROI : 1*DMN__LFPAR group : 1*ctrl -1*dual" \

-gltLabel 50 DMN__LFPAR--group\(+ctrl-mdd\) -gltCode 50 "ROI : 1*DMN__LFPAR group : 1*ctrl -1*mdd" \

-gltLabel 51 DMN__LFPAR--group\(+ctrl-ptsd\) -gltCode 51 "ROI : 1*DMN__LFPAR group : 1*ctrl -1*ptsd" \

-gltLabel 52 DMN__LFPAR--group\(+dual-mdd\) -gltCode 52 "ROI : 1*DMN__LFPAR group : 1*dual -1*mdd" \

-gltLabel 53 DMN__LFPAR--group\(+dual-ptsd\) -gltCode 53 "ROI : 1*DMN__LFPAR group : 1*dual -1*ptsd" \

-gltLabel 54 DMN__LFPAR--group\(+mdd-ptsd\) -gltCode 54 "ROI : 1*DMN__LFPAR group : 1*mdd -1*ptsd" \

-gltLabel 55 DMN__LFPAR--group^^ctrl -gltCode 55 "ROI : 1*DMN__LFPAR group : 1*ctrl " \

-gltLabel 56 DMN__LFPAR--group^^dual -gltCode 56 "ROI : 1*DMN__LFPAR group : 1*dual " \

-gltLabel 57 DMN__LFPAR--group^^mdd -gltCode 57 "ROI : 1*DMN__LFPAR group : 1*mdd " \

-gltLabel 58 DMN__LFPAR--group^^ptsd -gltCode 58 "ROI : 1*DMN__LFPAR group : 1*ptsd " \

-gltLabel 59 DMN__LFPAR--age -gltCode 59 "ROI : 1*DMN__LFPAR age : " \

-gltLabel 60 DMN__LFPAR--avgmot -gltCode 60 "ROI : 1*DMN__LFPAR avgmot : " \

-gltLabel 61 DMN__RFPAR--group\(+ctrl-dual\) -gltCode 61 "ROI : 1*DMN__RFPAR group : 1*ctrl -1*dual" \

-gltLabel 62 DMN__RFPAR--group\(+ctrl-mdd\) -gltCode 62 "ROI : 1*DMN__RFPAR group : 1*ctrl -1*mdd" \

-gltLabel 63 DMN__RFPAR--group\(+ctrl-ptsd\) -gltCode 63 "ROI : 1*DMN__RFPAR group : 1*ctrl -1*ptsd" \

-gltLabel 64 DMN__RFPAR--group\(+dual-mdd\) -gltCode 64 "ROI : 1*DMN__RFPAR group : 1*dual -1*mdd" \

-gltLabel 65 DMN__RFPAR--group\(+dual-ptsd\) -gltCode 65 "ROI : 1*DMN__RFPAR group : 1*dual -1*ptsd" \

-gltLabel 66 DMN__RFPAR--group\(+mdd-ptsd\) -gltCode 66 "ROI : 1*DMN__RFPAR group : 1*mdd -1*ptsd" \

-gltLabel 67 DMN__RFPAR--group^^ctrl -gltCode 67 "ROI : 1*DMN__RFPAR group : 1*ctrl " \

-gltLabel 68 DMN__RFPAR--group^^dual -gltCode 68 "ROI : 1*DMN__RFPAR group : 1*dual " \

-gltLabel 69 DMN__RFPAR--group^^mdd -gltCode 69 "ROI : 1*DMN__RFPAR group : 1*mdd " \

-gltLabel 70 DMN__RFPAR--group^^ptsd -gltCode 70 "ROI : 1*DMN__RFPAR group : 1*ptsd " \

-gltLabel 71 DMN__RFPAR--age -gltCode 71 "ROI : 1*DMN__RFPAR age : " \

-gltLabel 72 DMN__RFPAR--avgmot -gltCode 72 "ROI : 1*DMN__RFPAR avgmot : " \

-gltLabel 73 DMN__SAL--group\(+ctrl-dual\) -gltCode 73 "ROI : 1*DMN__SAL group : 1*ctrl -1*dual" \

-gltLabel 74 DMN__SAL--group\(+ctrl-mdd\) -gltCode 74 "ROI : 1*DMN__SAL group : 1*ctrl -1*mdd" \

-gltLabel 75 DMN__SAL--group\(+ctrl-ptsd\) -gltCode 75 "ROI : 1*DMN__SAL group : 1*ctrl -1*ptsd" \

-gltLabel 76 DMN__SAL--group\(+dual-mdd\) -gltCode 76 "ROI : 1*DMN__SAL group : 1*dual -1*mdd" \

-gltLabel 77 DMN__SAL--group\(+dual-ptsd\) -gltCode 77 "ROI : 1*DMN__SAL group : 1*dual -1*ptsd" \

-gltLabel 78 DMN__SAL--group\(+mdd-ptsd\) -gltCode 78 "ROI : 1*DMN__SAL group : 1*mdd -1*ptsd" \

-gltLabel 79 DMN__SAL--group^^ctrl -gltCode 79 "ROI : 1*DMN__SAL group : 1*ctrl " \

-gltLabel 80 DMN__SAL--group^^dual -gltCode 80 "ROI : 1*DMN__SAL group : 1*dual " \

-gltLabel 81 DMN__SAL--group^^mdd -gltCode 81 "ROI : 1*DMN__SAL group : 1*mdd " \

-gltLabel 82 DMN__SAL--group^^ptsd -gltCode 82 "ROI : 1*DMN__SAL group : 1*ptsd " \

-gltLabel 83 DMN__SAL--age -gltCode 83 "ROI : 1*DMN__SAL age : " \

-gltLabel 84 DMN__SAL--avgmot -gltCode 84 "ROI : 1*DMN__SAL avgmot : " \

-gltLabel 85 RFPAR__LFPAR--group\(+ctrl-dual\) -gltCode 85 "ROI : 1*RFPAR__LFPAR group : 1*ctrl -1*dual" \

-gltLabel 86 RFPAR__LFPAR--group\(+ctrl-mdd\) -gltCode 86 "ROI : 1*RFPAR__LFPAR group : 1*ctrl -1*mdd" \

-gltLabel 87 RFPAR__LFPAR--group\(+ctrl-ptsd\) -gltCode 87 "ROI : 1*RFPAR__LFPAR group : 1*ctrl -1*ptsd" \

-gltLabel 88 RFPAR__LFPAR--group\(+dual-mdd\) -gltCode 88 "ROI : 1*RFPAR__LFPAR group : 1*dual -1*mdd" \

-gltLabel 89 RFPAR__LFPAR--group\(+dual-ptsd\) -gltCode 89 "ROI : 1*RFPAR__LFPAR group : 1*dual -1*ptsd" \

-gltLabel 90 RFPAR__LFPAR--group\(+mdd-ptsd\) -gltCode 90 "ROI : 1*RFPAR__LFPAR group : 1*mdd -1*ptsd" \

-gltLabel 91 RFPAR__LFPAR--group^^ctrl -gltCode 91 "ROI : 1*RFPAR__LFPAR group : 1*ctrl " \

-gltLabel 92 RFPAR__LFPAR--group^^dual -gltCode 92 "ROI : 1*RFPAR__LFPAR group : 1*dual " \

-gltLabel 93 RFPAR__LFPAR--group^^mdd -gltCode 93 "ROI : 1*RFPAR__LFPAR group : 1*mdd " \

-gltLabel 94 RFPAR__LFPAR--group^^ptsd -gltCode 94 "ROI : 1*RFPAR__LFPAR group : 1*ptsd " \

-gltLabel 95 RFPAR__LFPAR--age -gltCode 95 "ROI : 1*RFPAR__LFPAR age : " \

-gltLabel 96 RFPAR__LFPAR--avgmot -gltCode 96 "ROI : 1*RFPAR__LFPAR avgmot : " \

-gltLabel 97 SAL__LFPAR--group\(+ctrl-dual\) -gltCode 97 "ROI : 1*SAL__LFPAR group : 1*ctrl -1*dual" \

-gltLabel 98 SAL__LFPAR--group\(+ctrl-mdd\) -gltCode 98 "ROI : 1*SAL__LFPAR group : 1*ctrl -1*mdd" \

-gltLabel 99 SAL__LFPAR--group\(+ctrl-ptsd\) -gltCode 99 "ROI : 1*SAL__LFPAR group : 1*ctrl -1*ptsd" \

-gltLabel 100 SAL__LFPAR--group\(+dual-mdd\) -gltCode 100 "ROI : 1*SAL__LFPAR group : 1*dual -1*mdd" \

-gltLabel 101 SAL__LFPAR--group\(+dual-ptsd\) -gltCode 101 "ROI : 1*SAL__LFPAR group : 1*dual -1*ptsd" \

-gltLabel 102 SAL__LFPAR--group\(+mdd-ptsd\) -gltCode 102 "ROI : 1*SAL__LFPAR group : 1*mdd -1*ptsd" \

-gltLabel 103 SAL__LFPAR--group^^ctrl -gltCode 103 "ROI : 1*SAL__LFPAR group : 1*ctrl " \

-gltLabel 104 SAL__LFPAR--group^^dual -gltCode 104 "ROI : 1*SAL__LFPAR group : 1*dual " \

-gltLabel 105 SAL__LFPAR--group^^mdd -gltCode 105 "ROI : 1*SAL__LFPAR group : 1*mdd " \

-gltLabel 106 SAL__LFPAR--group^^ptsd -gltCode 106 "ROI : 1*SAL__LFPAR group : 1*ptsd " \

-gltLabel 107 SAL__LFPAR--age -gltCode 107 "ROI : 1*SAL__LFPAR age : " \

-gltLabel 108 SAL__LFPAR--avgmot -gltCode 108 "ROI : 1*SAL__LFPAR avgmot : " \

-gltLabel 109 SAL__RFPAR--group\(+ctrl-dual\) -gltCode 109 "ROI : 1*SAL__RFPAR group : 1*ctrl -1*dual" \

-gltLabel 110 SAL__RFPAR--group\(+ctrl-mdd\) -gltCode 110 "ROI : 1*SAL__RFPAR group : 1*ctrl -1*mdd" \

-gltLabel 111 SAL__RFPAR--group\(+ctrl-ptsd\) -gltCode 111 "ROI : 1*SAL__RFPAR group : 1*ctrl -1*ptsd" \

-gltLabel 112 SAL__RFPAR--group\(+dual-mdd\) -gltCode 112 "ROI : 1*SAL__RFPAR group : 1*dual -1*mdd" \

-gltLabel 113 SAL__RFPAR--group\(+dual-ptsd\) -gltCode 113 "ROI : 1*SAL__RFPAR group : 1*dual -1*ptsd" \

-gltLabel 114 SAL__RFPAR--group\(+mdd-ptsd\) -gltCode 114 "ROI : 1*SAL__RFPAR group : 1*mdd -1*ptsd" \

-gltLabel 115 SAL__RFPAR--group^^ctrl -gltCode 115 "ROI : 1*SAL__RFPAR group : 1*ctrl " \

-gltLabel 116 SAL__RFPAR--group^^dual -gltCode 116 "ROI : 1*SAL__RFPAR group : 1*dual " \

-gltLabel 117 SAL__RFPAR--group^^mdd -gltCode 117 "ROI : 1*SAL__RFPAR group : 1*mdd " \

-gltLabel 118 SAL__RFPAR--group^^ptsd -gltCode 118 "ROI : 1*SAL__RFPAR group : 1*ptsd " \

-gltLabel 119 SAL__RFPAR--age -gltCode 119 "ROI : 1*SAL__RFPAR age : " \

-gltLabel 120 SAL__RFPAR--avgmot -gltCode 120 "ROI : 1*SAL__RFPAR avgmot : " \

-dataTable @group_w_age_avgmot_MVMtbl.txt
